# Modulation on Glutamic Pathway of Frontal-Striatum-Thalamus by rs11146020 and rs3813296 Gene Polymorphism in First-Episode Negative Schizophrenia

**DOI:** 10.3389/fnins.2020.00351

**Published:** 2020-04-21

**Authors:** Suping Cai, Yahui Lv, Kexin Huang, Wei Zhang, Qiang Wang, Liyu Huang, Jijun Wang

**Affiliations:** ^1^School of Life Sciences and Technology, Xidian University, Xi’an, China; ^2^The First Affiliated Hospital, Xi’an Jiaotong University, Xi’an, China; ^3^Shanghai Mental Health Center, Shanghai Jiao Tong University, Shanghai, China

**Keywords:** single nucleotide polymorphism, magnetic resonance imaging, glutamic pathway, causality connection, schizophrenia

## Abstract

**Objectives:**

The frontal-striatum-thalamus pathway is important in the glutamic neural circuit. The hypofunction of GRIN1 and GRIA2 subunits from glutamic receptors has been hypothesized as the primary process in the etiology of schizophrenia. Identified gene polymorphism involved in the pathogenesis of schizophrenia may uncover relevant mechanism pathways.

**Methods:**

We selected two loci of rs11146020 and rs3813296 distributed in GRIN1 and GRIA2 genes and tested their main and interaction effects on causality connections and structural characteristics in the frontal-striatum-thalamus pathway in 55 Han Chinese first-episode negative schizophrenia patients.

**Results:**

We found that: (1) rs11146020 has a significant main effect on the causality connections between the bilateral dorsolateral prefrontal cortex, and rs3813296 mainly influences those of the descending pathway from the prefrontal cortex to the striatum; (2) interaction effect of rs11146020 and rs3813296 on causality connections are located in the ascending pathway from the pallidum to the dorsolateral prefrontal cortex; and (3) the two loci have effects on the volumes of several regions of this pathway.

**Conclusion:**

Our results suggested there is modulation on glutamic frontal-striatum-thalamus pathway by rs11146020 and rs3813296 gene polymorphism. Patients with different genotypes have different neuroimaging characteristics, which indirectly reminded clinicians those patients should receive different clinical interventions.

## Introduction

Traditionally, abnormal dopamine has been considered the major underlying cause of schizophrenia (Gründer and Cumming, 2016). However, this conventional hypothesis does not readily elucidate the negative symptoms and cognitive deficits that are often observed in schizophrenia (Yang and Tsai, 2017). Glutamate, an important excitatory neurotransmitter in the brain, is necessary for neuronal growth, maturation, and synaptic plasticity (Garthwaite and Balazs, 1978; Choi, 1988; Derkach et al., 2007; Lau and Zukin, 2007). Previous studies have demonstrated that altered glutamate signaling may provide a better explanation for the pathological basis of schizophrenia (Stahl, 2007). One hypothesis declares that aberrant functioning of glutamatergic synapses leads to an imbalance between excitation and inhibition and, ultimately, to generate changes in the neural circuitry, such as in the frontal-striatum-thalamus pathway which is an important glutamic neural circuit, that drives psychosis and the impairment of cognitive functions (Schwartz et al., 2012).

N-methyl-D-aspartate (NMDA) and α-amino-3-hydroxy-5-methylisoxazole-4-propionic acid (AMPA) are two ionotropic receptors of glutamate that have been proposed as mediators of numerous common neuropsychiatric phenotypes such as cognition deficit, psychosis, and degeneration (Nakanishi et al., 1998). NMDA receptor is crucial for neuronal communication and the formation of tetrameric complexes of its was encoded by seven homologous subunits genes (Traynelis et al., 2010; Zhu et al., 2016). Although all are good candidate genes for the pathogenesis of schizophrenia, GRIN1 gene gets special attention, which codes NMDA receptor subunit 1 (NR1). Reducing expression of NR1 in mice gives rise to behavioral anomalies which is similar to those observed in pharmacologically induced animal models of schizophrenia (Mohn et al., 1999). rs11146020 is located in the 5′ untranslated region (UTR) in the GRIN1 gene, which may influence gene expression by affecting transcription, stability of mRNA, and translation efficiency (Meijer and Thomas, 2002; Wilkie et al., 2003). Liu et al. (2019) have illustrated that single nucleotide polymorphism (SNP) of rs11146020 in the promoter region of the GRIN1 gene are associated with schizophrenia in a Chinese Han population.

α-amino-3-hydroxy-5-methylisoxazole-4-propionic acid receptor is mainly located in excitatory synapses, where it mediates the most of fast synaptic transmission and participates in synaptic plasticity (Huganir and Nicoll, 2013). There are four AMPA receptor subunits assembled into functional homo- and hetero-tetrameric receptor complexes (Keinanen et al., 1990). Four genes (GRIA1-4) encode these receptor subunits which are expressed in several brain regions, such as the nucleus accumbens, striatum and prefrontal cortex (Reimers et al., 2011). Among the four receptor subunits, the Ca^2+^ permeability of AMPA receptors is dependent on the encoding of GRIA2 gene and AMPA receptors not containing the GRIA2 subunit are Ca^2+^ impermeable, increasing neuronal vulnerability to excitotoxicity, which results in neuropsychiatric symptoms (Isaac et al., 2007). GRIA2 is expressed on pyramidal cells and GABAergic interneurons, the cellular source of the expression difference would have a substantial effect on its physiological consequences (Lake et al., 2016). Moreover, The association of genotype rs3813296 T/T in the GRIA2 gene with a low efficacy of antipsychotics against negative symptoms was demonstrated when studying the association of polymorphisms of GRIA2 encoding a number of subunits of AMPA (Gareeva, 2018).

A previous study concluded that a series of glutamic neurons that begin in the prefrontal lobe connect and project into brainstem, midbrain, and limbic system (Schwartz et al., 2012). By this means, neurons originating in the prefrontal cortex may penetrate into deeper brain areas to control over midbrain neurons that are primarily in charge of creating and projecting neurotransmitter activities that are eventually responsible for drive and affective initiation. These deeper brain areas, such as the striatum and thalamus, play an important role on creating appropriate perceptual balance versus psychosis (Marx et al., 2015).

More importantly, twin and family studies indicated that genetic factors contributed substantially to the possibility of developing schizophrenia (Ripke et al., 2014). The extant data suggested that schizophrenia involved complex interactions between multiple genes, each exerting relatively small effects on vulnerability. Several SNPs have been associated with increased risk for developing schizophrenia, although few of these findings have been replicated (Jagannath et al., 2018). If confirmed in additional studies, these genetic markers would implicate glutamatergic neurotransmitter pathways in the pathogenesis of schizophrenia (Dauvermann et al., 2017; Saini et al., 2017; Han et al., 2018).

Taken together, the modulation relationship between the SNP variants in specific genes and glutamic neural circuits remains a challenge to understand. If we obtained the association relationship between them, it could potentially help clinicians regulate intervention strategies for those patients with some genotypes. Based on this challenge, in the present study, we selected an important glutamic neural circuit, the frontal-striatum-thalamus pathway, and two SNPs in GRIN1 and GRIA2 genes to explore their effects on the causality connections and structural characteristics of this neural pathway and then investigated the correlation between the causality connection strength and clinical cognitive behavioral scores. The hypothesis is that there is a modulation on glutamic pathway of frontal-striatum-thalamus by rs11146020 and rs3813296 gene polymorphism in first-episode negative schizophrenia.

## Materials and Methods

### Participant Selection

We selected fifty-five first-episode negative schizophrenic patients from the Shanghai Mental Health Center. All patients met the inclusion criteria as follows: (1) they were first-episode and had no medication history; (2) they were diagnosed as schizophrenia by senior clinical psychiatrists using a structural clinical interview from the DSM-IV-TR (patient edition); (3) they did not present severe agitation or aggression; and (4) they were 18–45 years old and right-handed.

### Behavioral Measurement Scales

The patients were assessed using scales for the assessment of negative symptoms (SANS), duration of untreated psychosis (DUP), and education years (EDU). Intravenous peripheral blood of each patient was drawn for the extraction genotype. More importantly, the internationally recognized consensus version of cognitive function tests for schizophrenia were measured, including the trail making test (TMT); brief assessment of cognition in schizophrenia-symbol coding (BACS-SC); verbal fluency (VF); continuous performance test-identical pairs (CPT-IP); Wechsler memory scale, third edition: spatial span (WMS-III SS); Hopkins verbal learning test, revised (HVLT-R); brief visuospatial memory test, revised (BVMT-R); neuropsychological assessment battery, mazes (NAB-M); and Mayer-Salovey-Caruso emotional intelligence test, managing emotions (MSCEIT-ME). All tests were confirmed by three experienced psychiatrists who underwent consistency training for approximately 1 week, and all scores were assessed objectively. For more detailed information, please see Table 1.

**TABLE 1:** Genotypic, demographic, and clinical information of all participants.

SNP ID	Alleles	Location	Call Rate (%)	Test for HWE (*P* Value)	MAF	
**GRIN1**						
rs11146020	C/G	upstream 9:137138632	100	0.6142^a^	C: 0.195	
**GRIA2**						
rs3813296	G/T	Intron 4:157360371	100	0.1999^a^	G:0.246	

**rs11146020**	**GG = 31**		**CG = 22**		***F* value**	***P* value**
**rs3813296**	**GT = 14**	**TT = 17**	**GT = 10**	**TT = 12**		

Gender	6M/8F	8M/9F	6M/54F	7M/5F	1.14	0.79^b^
AGE	26.67 ± 6.08	23.87 ± 5.85	25.11 ± 7.41	25.11 ± 5.04	0.47	0.71^c^
EDU	12.42 ± 3.80	12.07 ± 2.81	12.78 ± 2.68	13.56 ± 2.40	0.48	0.70^c^
Head motion	0.36 ± 0.21	0.44 ± 0.24	0.29 ± 0.20	0.38 ± 0.19	0.51	0.63^c^
DUP	38.42 ± 44.01	26.47 ± 16.08	24.22 ± 18.44	36.89 ± 47.96	0.50	0.68^c^
SANS	14.75 ± 11.38	14.40 ± 13.14	14.00 ± 10.42	24.67 ± 13.30	1.69	0.18^c^
TMT	46.08 ± 18.83	31.07 ± 18.10	40.89 ± 16.10	52.56 ± 24.98	2.63	0.06^c^
BACS-SC	51.75 ± 10.06	52.33 ± 13.07	47.89 ± 14.22	47.56 ± 12.07	0.45	0.72^c^
HVLT-R	22.08 ± 5.18	24.00 ± 7.00	20.56 ± 6.15	24.00 ± 4.80	0.80	0.50^c^
WMS-III SS	14.58 ± 2.78	13.93 ± 4.51	13.11 ± 3.44	15.11 ± 3.66	0.50	0.68^c^
NAB-M	15.00 ± 7.06	14.13 ± 5.96	11.66 ± 7.90	12.22 ± 9.52	0.47	0.71^c^
BVMT-R	23.17 ± 7.25	25.27 ± 9.19	21.89 ± 8.40	20.44 ± 9.88	0.65	0.59^c^
VF	20.08 ± 6.65	18.53 ± 6.00	19.33 ± 5.98	23.56 ± 7.84	1.16	0.34^c^
MSCEIT-ME	86.92 ± 13.19	84.93 ± 15.11	81.56 ± 15.11	96.67 ± 19.98	1.58	0.21^c^
CPT-IP	2.42 ± 1.08	1.80 ± 1.01	2.33 ± 0.87	2.22 ± 0.83	1.07	0.37^c^
Head motion	0.34 ± 0.07	0.29 ± 0.06	0.37 ± 0.09	0.33 ± 0.08	0.70	0.55^c^

*Data are given as mean ± standard deviation; ^*a*^*P* value was obtained by hardy-weinberg equilibrium (HWE) test; ^*b*^*P* value was obtained by an independence Pearson chi-square test. ^*c*^The *P* and corresponding *F* values were obtained by a one-way analysis of variance test. MAF, minor allele frequency; EDU, education years; DUP, duration of untreated psychosis; SANS, assessment of negative symptoms; TMT, trail making test; BACS-SC, brief assessment of cognition in schizophrenia-symbol coding; HVLT-R, Hopkins verbal learning test, revised; WMS-III SS, Wechsler memory scale, third edition: spatial span; NAB-M, neuropsychological assessment battery, mazes; BVMT-R, brief visuospatial memory test, revised; VF, verbal fluency; MSCEIT-ME, Mayer-Salovey-Caruso emotional intelligence test, managing emotions; CPT-IP, continuous performance test-identical pairs.*

### Genotyping

Peripheral blood was drawn from a participant’s vein into a sterile tube containing EDTA. We stored the plasma samples at −80°C. Genomic DNA was isolated from peripheral blood leukocytes according to the manufacturer’s protocol (Thermo Fisher Scientific, United States). DNA was also stored at −80°C for SNP analysis. Genotyping was performed for all SNPs by SnaPshot using a 3730xl DNA Analyzer (Thermo Fisher Scientific, United States).

Single nucleotide polymorphism rs11146020 from the GRIN1 gene and rs3813296 from the GRIA2 gene were genotyped in all patients by allele-specific polymerase chain reaction primers. The success rate of the genotyping in our study was 100%. Information from the Hardy-Weinberg equilibrium (HWE) and minor allele frequency (MAF) are shown in Table 1. In addition, we have uploaded the SNP data to a publicly available repository^1^. The link to the SNP data is https://www.synapse.org/#!Synapse:syn21788916/tables/.

There were two genotypes for rs11146020 among the 55 schizophrenia patients: CG (23 patients) and GG (32 patients). Analogously, there were three genotypes for rs3813296: GT (24 patients), TT (29 patients), and GG (2 patients). Because only two participants were the carriers of genotype GG, we did not select these participants. To further investigate the interaction effect of rs11146020 and rs3813296, we divided the patients into four subgroups (14 GG/GT, 17 GG/TT, 10 CG/GT, and 12 CG/TT) for follow-up analysis.

### Data Acquisition

All MRI images were scanned using a 3T Siemens Magnetom Verio Syngo MR B17 scanner. Participants were informed to keep their eyes closed, not to focus their thoughts on anything and stay awake.

The parameters of functional MRI data are as follows: echo time [TE] = 30 ms, repetition time [TR] = 3 s, flip angle [FA] = 90°, slice thickness = 3.0 mm, slices = 45, field of view [FOV] = 220 mm × 220 mm, matrix size = 64 × 64, voxel size = 3 mm × 3 mm × 3 mm and 170 slices.

Structural MRI data were obtained with a high-resolution T1-weighted magnetization-prepared rapid gradient echo (MPRAGE) sequence. The parameters used are as follows: TE = 2.56 ms, TR = 2530 ms, FA = 7°, FOV = 256 mm × 256 mm, matrix = 256 × 256, slice thickness = 1 mm, inversion time = 1100 ms, and 192 coronal slices.

### Data Preprocessing

#### T1-Weighted Data Preprocessing

We performed T1-weighted data processing with the FSL-VBM protocol with the FMRIB Software Library 4.1 (FSL^2^). More detailed processing, please see our recent study (Cai et al., 2017a). There were four steps: brain extraction; the segmentation of white matter, gray matter and cerebrospinal fluid; image registration to the standard template; and image smoothing with a Gaussian kernel with 8 mm.

We segmented white matter and gray matter and divided the images into four subgroups based on different genotypes (GG/GT, GG/TT, CG/GT, and CG/TT) for follow-up statistical analysis.

#### fMRI Data Preprocessing

fMRI data processing was performed using a MATLAB toolbox called DPABI (Yan et al., 2016), which evolved from REST (Song et al., 2011), and DPARSF (Chao-Gan and Yu-Feng, 2010). For more details on fMRI data processing, please see our previous study (Cai et al., 2015). There were eight steps: discarding the first ten time points, slice timing correction, correcting for head motion (exclusion criteria: exceeding 1.5 mm in any dimension of x, y, and z or 1.5° in any angular motion; two participants were removed), normalizing to individual T1-weighted anatomical images, smoothing images, removing linear trends, filtering (0.01–0.1 Hz) and regressing the covariates (A Friston-24 parameter, the global mean signal, cerebrospinal fluid signal, and white matter signal were the nuisance covariates) (Friston et al., 1996; Yan et al., 2013). We also examined if there are any differences in head motion among groups as described in the study of Power et al. (2012).

### Data Processing and Statistical Analysis

#### Frontal-Striatum-Thalamus Pathway and Core Regions Selection

**FIGURE 1:**
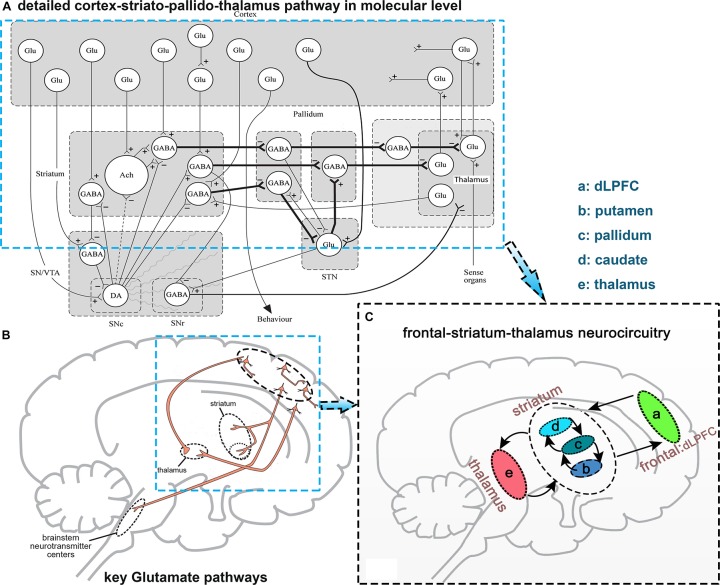
Diagram explaining the selection of the frontal-striatum-thalamus pathway. **(A)** Detailed cortex-striato-pallido-thalamus pathway at the molecular level, this figure is from Carlsson et al. (1999); **(B)** several key glutamic pathways in synaptic release level, this figure from Schwartz et al. (2012); **(C)** selected frontal-striatum-thalamus pathway. Abbreviations: Glu, Glutamate; DA, dopamine; GABA, gamma aminobutyric acid.

At a molecular level, glutamate neurotransmitter release and synaptic discharge circuit are mainly from the frontal lobe, and go through the striatum to the basal ganglia (Carlsson et al., 1999; Schwartz et al., 2012; Figures 1A,B). Based on these studies, we concluded and focused on one of the glutamic neurotransmission pathways: the frontal-striatum-thalamus pathway (Figure 1) and selected 10 core brain regions in this pathway: left and right dorsolateral prefrontal cortex (L/R. dLPFC), L/R. caudate, L/R. putamen, L/R. pallidum, and L/R. thalamus (Schwartz et al., 2012). We extracted these brain regions using an anatomical automatic labeling (AAL) template implemented with REST (Song et al., 2011). It should be noted that dLPFC is a functional definition, thus we used dorsolateral superior frontal gyrus to represent this region based on the AAL template. Ten brain regions were resampled to the spatial resolution of fMRI images.

#### Granger Causality Analysis

Granger causality analysis (GCA) is an approach used to explore the dynamic causal relationship between two time series (Granger, 1969). A brief introduction of the Granger procedure is provided here. For two given fMRI time series x(t) and y(t), x(t) is the Granger causing y(t) if the past information of x(t) can improve the prediction of the current value of y(t). The Granger causal relationship between the two series is often estimated by vector autoregressive (VAR) modeling. Granger causality can evaluate the direct linear influence from x(t) to y(t) (F_*XtoY*_) and the linear direct influence from y(t) to x(t) (F_*YtoX*_). Formula (1) is the mathematic model of GCA:

$$x\,\left(t\right)=\alpha_{x,0}+\sum^{p}\alpha_{x\,x,i}\,x\,\left(t-i\right)+\sum^{p}\alpha_{x\,y,i}\,y\,\left(t-i\right)+\sum^{q}\beta_{x,i}\,z_{j}\,\left(t\right)+\varepsilon_{x}\,\left(t\right)$$

(1) $$y\,\left(t\right)=\alpha_{y,0}+\sum^{p}\alpha_{y\,x,i}\,x\,\left(t-i\right)+\sum^{p}\alpha_{y\,y,i}\,y\,\left(t-i\right)+\sum^{q}\beta_{y,i}\,z_{j}\,\left(t\right)+\varepsilon_{y}\,\left(t\right)$$

where *z_*j*_(t)* represents up to *q* exogenous processes (six orthogonal motion estimates and physiological noise) independent of the path network (*j* = 1,…., *q*). Contributions of each lagged variable to the prediction of its respective target are denoted by α; β corresponds to the covariate effect, and prediction errors of individual models are denoted by ε. A similar method was used in one of our previous studies (Cai et al., 2017b).

We applied bivariate coefficient-based GCA to compute the causality of the 10 brain regions for each participant. The average time courses of each region were input to the GCA using REST (Song et al., 2011). Then, we obtained the path coefficients characterized by the direction and the strength of the temporal relation among the 10 brain regions.

#### Main and Interaction Effect Analysis of rs11146020 and rs3813296

To understand the main and interaction effects of rs11146020 and rs3813296 on the frontal-striatum-thalamus pathway, we performed multivariable general linear model (GLM) analysis with gender, EDU, and age as regressors using IBM SPSS Statistical 23. There are two main effect analyses of rs11146020 and rs3813296 and one interaction effect analysis of rs11146020^∗^rs3813296 in 90 ($A_{10}^{2}$) causality connections. If there are significant interactions, simple effect analysis was conducted with a script embedded into IBM SPSS Statistical 23. We applied the Mann–Whitney *U* test to test significance and false discovery rate (FDR) to correct the multiple comparisons (*P* < 0.01).

#### Gray and White Matter Structural Analysis

To investigate the differences between four subgroups (GG/GT, GG/TT, CG/GT, and CG/TT) in the gray and white matter structure, we applied two-sample *t*-tests to assess structural alterations in gray and white matter with gender, ages and EDU as regressors. The statistical images were corrected by FDR for multiple comparisons correction (*P* < 0.01).

### Correlation Analysis Between Connection Strength and Behavioral Scores

The Spearman test for correlation was applied to investigate the correlation between connection strength, which showed significant group difference, and clinical scales, including TMT, BACS-SC, VF, CPT-IP, WMS-III SS, HVLT-R, BVMT-R, NAB-M, and MSCEIT-ME. The significance levels were set at *P* < 0.01 (two-tailed, FDR correction).

## Results

### No Significant Subgroup Difference in Demographic Information and Clinical Scores

No significant difference were found in gender, age, gender, head motion, EDU, DUP, SANS, TMT, BACS-SC, VF, CPT-IP, WMS-III SS, HVLT-R, BVMT-R, NAB-M, and MSCEIT-ME among the four subgroups (*P* > 0.05). There is no difference in head motion among groups. All demographic information and clinical scores are shown in Table 1.

### Main and Interaction Effects on Causality Connectivity in the Frontal- Striatum-Thalamus Pathway

**FIGURE 2:**
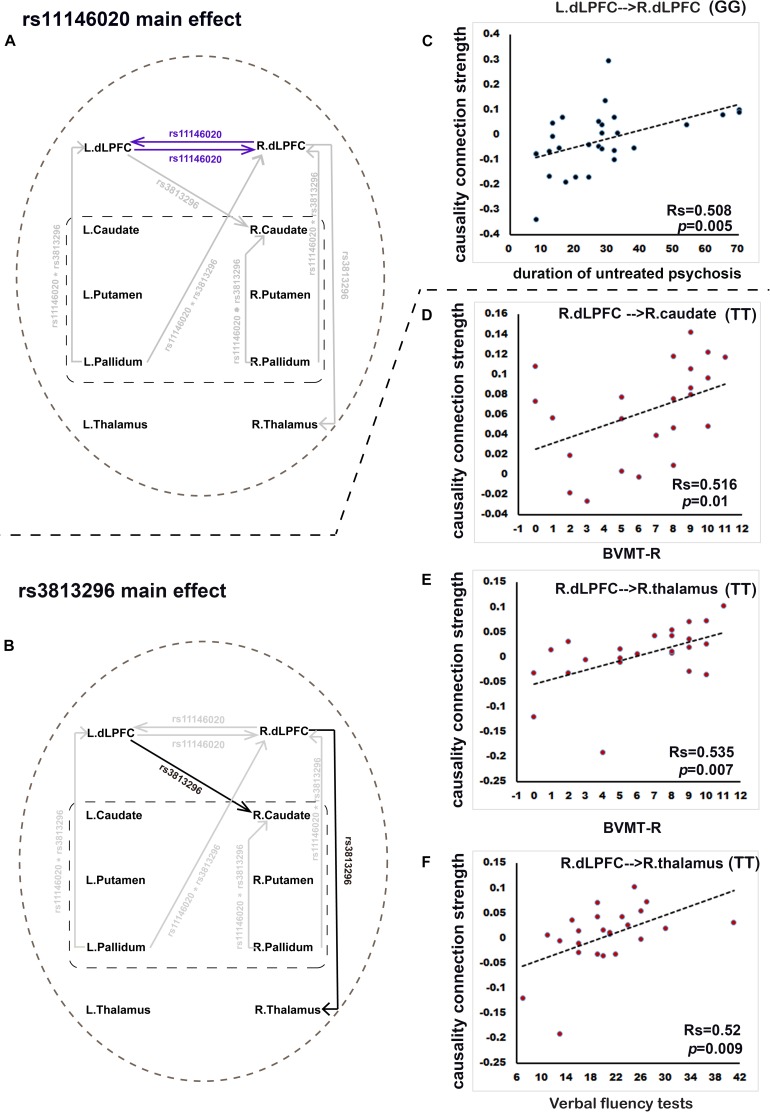
Rs11146020 and rs3813296 main effects on causality connections and relationships with behavioral variables (*P* < 0.01, FDR correction). **(A)** rs11146020 and **(B)** rs3813296 main effects on causality connections; **(C)** causality connection strength of L. dLPFC → R. dLPFC has significant positive correlation with duration of untreated psychosis in patients with the GG genotype; **(D)** causality connection strength of R. dLPFC → R. caudate has a significant positive correlation with BVMT scores in patients with the TT genotype; causality connection strength of R. dLPFC → R. thalamus has a significant positive correlation with BVMT-R scores **(E)** and verbal fluency test scores **(F)** in patients with the TT genotype. Abbreviations: dLPFC, dorsolateral prefrontal cortex; BVMT-R, brief visuospatial memory test, revised.

Rs11146020 mainly affects the causality connectivity within the dLPFC: L. dLPFC → R. dLPFC and L. dLPFC → R. dLPFC (Figure 2A and Table 2). Rs3813296 mainly affects the causality connectivity of the descending pathway from the dorsolateral prefrontal cortex to thalamus and striatum: L. dLPFC → R. caudate and R. dLPFC → R. thalamus (Figure 2B and Table 2).

**TABLE 2:** Main and interaction effects of rs11146020 and rs3813296 on causality connectivity in frontal-striatum-thalamus pathway.

rs11146020 main effect on causality connectivity in frontal- striatum- thalamus pathway				
GG vs. CG				
Causality connectivity	Connection strength		*F*(1) value	*P* value
	GG	CG		
L.dLPFCR.dLPFC	−0.028 ± 0.022	0.047 ± 0.027	8.29	0.006
R.dLPFCL.dLPFC	0.02 ± 0.024	−0.085 ± 0.03	7.539	0.009
**rs3813296 main effect on causality connectivity in frontal- striatum- thalamus pathway**				
**GT vs. TT**				
**Causality connectivity**	**Connection strength**		***F*(1) value**	***P* value**
	**TT**	**GT**		

L.dLPFCR.Caudate	0.056 ± 0.016	0.015 ± 0.015	7.535	0.009
R.dLPFCR.Thalamus	0.008 ± 0.012	0.026 ± 0.013	9.147	0.003
rs11146020*rs3813296 inteaction effect on causality connectivity in frontal- striatum- thalamus pathway				
**CG/GT vs. GG/GT**				
**Causality connectivity**	**Connection strength**		***F*(1) value**	***P* value**
	**GG/GT**	**CG/GT**		

L.PallidumL.dLPFC	−0.032 ± 0.06	0.116 ± 0.071	8.691	0.0043
L.PallidumR.dLPFC	−0.078 ± 0.051	0.096 ± 0.06	9.487	0.0024
**CG/TT vs. GG/GT**				
**Causality connectivity**	**Connection strength**		***F*(1) value**	***P* value**
	**GG/GT**	**CG/TT**		

R.PallidumR.dLPFC	−0.08 ± 0.057	0.169 ± 0.068	9.366	0.0029
R.PallidumR.Caudate	0.036 ± 0.026	0.134 ± 0.025	8.662	0.0044

*Data are given as mean ± standard deviation; *P* and corresponding *F* values were obtained from multivariable GLM analysis by applying the Mann–Whitney *U* test and FDR correction (*P* < 0.01). GLM, general linear model; FDR, false discovery rate; dLPFC, dorsolateral prefrontal cortex.*

**FIGURE 3:**
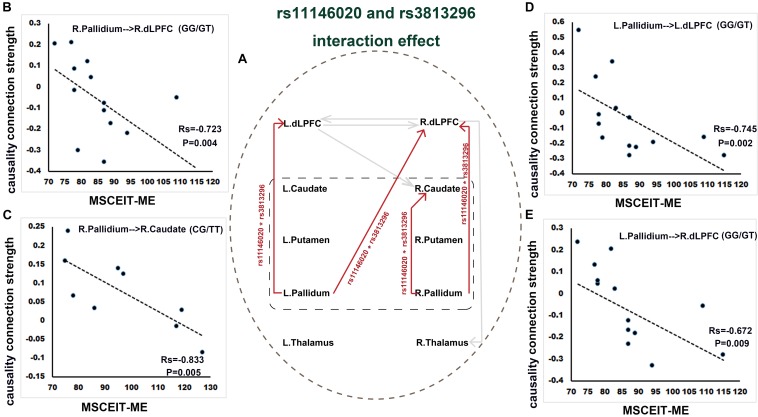
Interaction effect of rs11146020 and rs3813296 on causality connections and relationship with behavioral variables (*P* < 0.01, FDR correction). **(A)** interaction effect of rs11146020 and rs3813296 on causality connections; causality connection strength of R. pallidum → R. dLPFC **(B)**, L. pallidum → L. dLPFC **(D)** and L. pallidum → R. dLPFC **(E)** has a significant negative correlation with MSCEIT-ME in patients with the GG/GT genotype; causality connection strength of R. pallidum → R. caudate **(C)** has a significant negative correlation with MSCEIT-ME in patients with the CG/TT genotype; Abbreviations: dLPFC, dorsolateral prefrontal cortex; MSCEIT-ME, Mayer-Salovey-Caruso emotional intelligence test (managing emotions).

Rs11146020 and rs3813296 interactively affect the information flow of the upstream pathway from striatum to dLPFC: L. pallidum → L. dLPFC, L. pallidum → R. dLPFC, R. pallidum → R. dLPFC, and R. pallidum → R. caudate (Figure 3A and Table 2).

### Main and Interaction Effects on Gray and White Matter Structures

**FIGURE 4:**
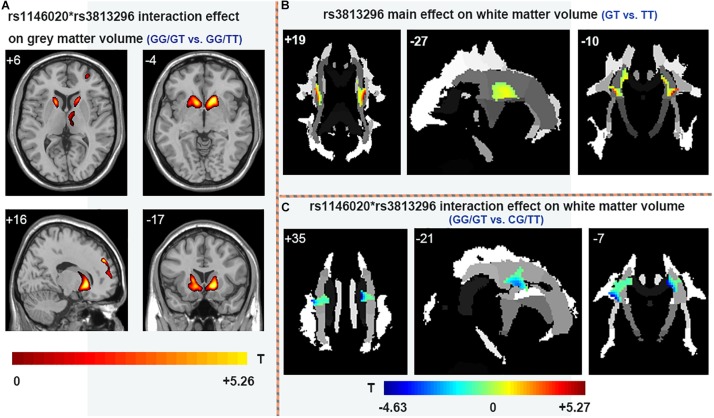
Main and interaction effects of rs11146020 and rs3813296 on gray and white matter (*P* < 0.01, FDR correction). **(A)** rs11146020 and rs3813296 interaction effect on gray matter volume (GG/GT vs. GG/TT); **(B)** rs3813296 main effect on white matter volume (GT vs. TT); **(C)** rs11146020 and rs3813296 interaction effect on white matter volume (GG/GT vs. CG/TT).

There is no main effect of rs11146020 on gray and white matter volumes and no main effect of rs3813296 on gray matter volumes. Main effect of rs3813296 on white matter were located in left/right superior corona radiata fiber (Figure 4B and Table 3) (*P* < 0.01, FDR correction). The interaction effect of rs3813296^∗^rs11146020 on gray and white matter volumes were located in the left/right putamen, left/right caudate, left/right thalamus (Figure 4A and Table 3) and left/right superior corona radiata fiber (Figure 4C and Table 3), respectively (*P* < 0.01, FDR correction).

**TABLE 3:** Main and interaction effects of rs11146020 and rs3813296 on gray and white matter volumes.

rs11146020*rs3813296 inteaction effect on gray matter volumes					
GG/GT vs. GG/TT					
Brain region	MNI			*T* value	*P* value
	X	Y	Z		
L.Caudate/thalamus	−15	14	6	4.563	0.005
R. Caudate/thalamus	17	17	6	4.288	0.005
L.Putamen/caudate	−9	13	−4	4.892	0.004
R. Putamen/caudate	16	17	−4	5.184	0.002
**rs3813296 main effect on white matter volumes**					
**GT vs. TT**					
**Brain region**	**MNI**			***T* value**	***P* value**
	**X**	**Y**	**Z**		

L. Superior corona radiata	−26	−3	21	3.681	0.008
R. Superior corona radiata	26	−8	32	3.455	0.010
rs11146020*rs3813296 inteaction effect on white matter volumes					
**GG/GT vs. CG/TT**					
**Brain region**	**MNI**			***T* value**	***P* value**
	**X**	**Y**	**Z**		

L. Superior corona radiata	−16	−6	35	−3.47	0.009
R. Superior corona radiata	29	−10	35	−3.394	0.010

**P* and corresponding *T* values were obtained by two samples *t*-test (*P* < 0.01, FDR correction). X, Y, and Z from MNI (Montreal Neurological Institute) coordinates.*

### Significant Correlation Between Causality Connection Strength and Behavioral Scales

We found that the strength of causality connection L. dLPFC → R. dLPFC has a significant positive correlation with DUP (Figure 2C and Table 4); the connection strengths of R. dLPFC → R. caudate and R. dLPFC → R. thalamus have significant positive correlations with BVMT-R scores (Figures 2D,E and Table 4); and the connection strength of R. dLPFC → R. thalamus has a significant positive correlation with VF test scores (Figure 2F and Table 4). More interestingly, four ascending causality connections interactively effected by rs11146020 and rs3813296 were all significant negative correlation with MSCEIT-ME scores (Figures 3B–E and Table 4).

**TABLE 4:** Significant correlation between causality connection strength and behavioral scales.

Causality connectivity	Genotype	SNP	Clinical scales	Rs value	*P* value
L.dLPFC → R.dLPFC	GG	rs11146020	DUP	0.508	0.005
R.dLPFC → R.Caudate	TT	rs3813296	BVMT-R	0.516	0.01
R.dLPFC → R.Thalamus	TT	rs3813296	BVMT-R	0.535	0.007
R.dLPFC → R.Thalamus	TT	rs3813296	VF	0.52	0.009
R.Pallidum → R.dLPFC	GG/GT	GG:rs11146020 GT:rs3813296	MSCEIT-ME	–0.723	0.004
R.Pallidum → R.Caudate	CG/TT	CG:rs11146020 TT:rs3813296	MSCEIT-ME	–0.833	0.005
L.pallidum → L.dLPFC	GG/GT	GG:rs11146020 GT:rs3813296	MSCEIT-ME	–0.745	0.002
L.Pallidum → R.dLPFC	GG/GT	GG:rs11146020 GT:rs3813296	MSCEIT-ME	–0.672	0.009

*Rs and *P* values were obtained from Spearman rank correlation test, *P* < 0.01, FDR correction. dLPFC, dorsolateral prefrontal cortex; DUP, duration of untreated psychosis; BVMT-R, brief visuospatial memory test, revised; VF, verbal fluency; MSCEIT-ME, Mayer-Salovey-Caruso emotional intelligence test, managing emotions.*

## Discussion

The hypo-function of GRIN1 and GRIA2 subunits from glutamic receptors has been hypothesized as a primary process in the pathophysiology of schizophrenia. Identified gene polymorphism involved in the etiology of schizophrenia may reveal relevant mechanistic pathways. Whether the polymorphisms in the subunit genes of GRIN1 and GRIA2 receptors contribute to the risk of schizophrenia is still in question. In our study, we selected two SNPs distributed in GRIN1 and GRIA2 genes and tested their effects on the causality connections and structural characteristics of the frontal-striatum-thalamus pathway in Han Chinese schizophrenia patients. There were three major findings: (1) rs11146020 has a significant main effect on the causality connections between the left and right dLPFC and rs3813296 mainly influences the descending pathway from the prefrontal lobe to the striatum; (2) the interaction effect of rs11146020 and rs3813296 is mainly located in the ascending pathway from the bilateral pallidum to the right caudate and the bilateral dLPFC; and (3) the two SNPs have main and interaction effects on the volumes of gray and white matter in several regions of this pathway. Some causality connection strengths affected by the two SNPs have remarkable correlation with clinical cognitive performances in VF, visuospatial memory and emotion management. The detailed explanation is as follows.

### Main Effect of rs11146020 on Causality Connectivity in Frontal-Striatum-Thalamus Pathway

Rs11146020 has a significant main effect on the causality connections between the left and right dLPFC. The ancestral and variant alleles of rs11146020 are G and C, respectively. There is a significant difference between homozygous GG and heterozygous GC in schizophrenia patients, indicating that genotype influences functional connections. Zhao et al. (2006) found that the C allele was expressed in a high frequency in schizophrenia patients. rs11146020 is located in the GRIN1 gene, which is a subunit of NMDAR. The gene responsible for its expression is located at 9q34 in the promoter region (Begni et al., 2003). The GRIN1 gene product plays a foundational role in many brain functions, and its involvement in the pathogenesis of schizophrenia has been widely investigated (Hung et al., 2002; Zhao et al., 2006). Moreover, GRIN1 knockout animals showed abnormal behavior characteristics which were commonly similar with patients with schizophrenia, such as impairment of working memory, reduced “self-care” (nest building) and social activity (Tatard-Leitman et al., 2015). Gray et al. (2015) reported higher expression levels of the majority of glutamatergic genes, especially GRIN1, were detected in the dLPFC. The rs11146020 genotype has a significant association with the causality connection between the left and right dLPFC, which is opportunely proved the suggestion by Zhao et al. (2006) that rs11146020 is a potential candidate to alter the risk of schizophrenia and worth further replication and functional investigation.

Interestingly, causality connection between the left and right dLPFC in schizophrenia patients with ancestral genotype GG has a significant positive correlation with DUP but was not found in GC carriers. For patients who carried the GG genotype, Increased connection strength could lead to protracted DUP in patients who carried the GG genotype. Research shows that there is a close relationship between longer DUP and poorer outcomes in first-episode psychosis (Rubio and Correll, 2017). Hence, enhanced connectivity between the left and right dLPFC is not an optimistic phenomenon, especially in patients with the GG genotype.

In terms of brain structure, we did not find a remarkable difference in the volumes of gray and white matter between GG and GC genotypes, suggesting that the association between genotype and function was unrelated to cortex and subcortex sizes. This is possibly because the mutation from allele G to C is not enough to change brain volume.

### Main Effect of rs3813296 on Causality Connectivity in the Frontal-Striatum-Thalamus Pathway

Rs3813296 mainly influences the causality connections of the descending pathway from the prefrontal lobe to the striatum, including L. dLPFC → R. caudate and R. dLPFC → R. thalamus. The connection strength of L. dLPFC → R. caudate in schizophrenia patients with the GT genotype is significantly lower than those with the TT genotype. Rs3813296 is located in the GRIA2 gene which is one subunit of the AMPA receptor (Lu et al., 2009). The Ca^2+^ permeability of AMPA receptors rely on the GRIA2 subunit and AMPA receptors without the GRIA2 subunit are Ca^2+^ impermeable, which increases the neuronal vulnerability to excitotoxicity and can result in neuropsychiatric symptoms (Isaac et al., 2007). The lower connection strength of L. dLPFC → R. caudate in patients with the GT genotype may be due to a deficit in Ca^2+^ permeability from cortex to subcortex. Based on the current result regarding to rs3813296, deficit Ca^2+^ permeability should be associated with the variation of T to G. However, this variation has significant relevance to the enhanced connection strength of R. dLPFC → R. thalamus, which could be interpreted as compensation phenomenon.

A literature study returned two studies containing rs3813296 (Crisafulli et al., 2012; Iamjan et al., 2018). Few studies are similar with our study; thus, we tried to search for supporting results from the current dataset and found that the white matter volume of the superior corona radiata in schizophrenia patients with the GT genotype is significantly larger than those with the TT genotype. Corona radiata are the most prominent projection fibers, and they are afferents that carry information to the cerebral cortex and efferent that carry information away from it (Morecraft et al., 2002). Gray matter of the striatum and white matter of the corona radiata are the main components of the basal ganglia. The difference of the white matter volume of the superior corona radiata between GT and TT genotypes explains the lower connection strength of L. dLPFC → R. caudate in patients with the GT genotype: inflated volume of the superior corona radiata indirectly causes dispersive connection strength between the two regions.

Causality connection strengths of L. dLPFC → R. caudate and R. dLPFC → R. thalamus in patients with the TT genotype have a significant positive correlation with scores in VF and brief visuospatial memory tests. The functional anatomy of VF has been well characterized in normal participants using positron emission tomography (PET) (Spence et al., 1998). The study also demonstrated that generating words beginning with a given letter activates the dLPFC. Another study suggested that the thalamus is involved in the encoding of verbal material and that thalamic damage impairs verbal recall (Ruggeri, 2016). Benson et al. (2008) hypothesized that cognitive impairments could be related to the dysfunction of the physiological metabolic activity between the dLPFC and subcortical regions. In particular, hypoactivation of the dLPFC observed in schizophrenia patients could result in hyperactivation of subcortical structures, such as the striatum and thalamus (Groerewegen, 1991). Thus, the significant correlation suggested that VF and brief visuospatial memory tests scores have important clinical significance. As suggested in previous studies, it is also closely associated with the degeneration of the brain in schizophrenia patients (Zhang et al., 2012, 2014; Cetin-Karayumak et al., 2019). Further, recent research mentioned that words and visuospatial memory information are conveyed across aforesaid regions via the excitatory projections of glutamatergic pyramidal neurons (Hoftman et al., 2017; Nikolova et al., 2017). More importantly, the current results are especially helpful for reminding clinical researchers to pay attention to the first-episode negative schizophrenia patients with the TT genotype. These patients are vulnerable to VF and visual spatial memory. In the long run, patients with the TT genotype have different characteristic and should be treated using different clinical interventions.

### Interaction Effect of rs11146020^∗^rs3813296 on Causality Connectivity in the Frontal-Striatum-Thalamus Pathway

The interaction effects of rs11146020^∗^rs3813296 on the causality connection are mainly located in the ascending pathway from the bilateral pallidum to the right caudate and bilateral dLPFC. After the interaction effect of the two SNPs, simple effect tests showed that modulation by rs11146020 on the causality connection of L. pallidum → R. dLPFC and L. pallidum → R. dLPFC is influenced by the GT genotype in rs3813296. Similarly, modulation by rs3813296 on the causality connection of R. pallidum → R. caudate is influenced by the GG genotype in rs11146020. Moreover, the interaction effect of the two SNP is also on the gray and white matter volumes of several regions in this pathway, such as parts of the caudate, thalamus, putamen and fiber of the superior corona radiata. Taken together, these results imply that the effect of two SNPs on brain structure and function is greater than that of a single SNP. It is consistent with a comment provided by previous research that suggested that the risk of common diseases is potentially determined by the complex interaction between genetic factors, including SNPs (Lohmueller et al., 2003; Mechanic et al., 2008).

Moreover, four causality connections, among those mentioned, have a significant negative correlation with MSCEIT-ME scores. To understand the negative correlation between them, we investigated the literature and examined gamma aminobutyric acid (GABA) interneurons, which are purported to ultimately inhibit the generation of excessive mesolimbic dopamine activity (Stahl, 2007). Disturbances in GABA neurotransmission could represent a common pathophysiology for different domains of cortical dysfunction in schizophrenia (Hashimoto et al., 2008). For example, if the GABA interneuron was dysfunctional, it would lead to excessive suppression and produce decreasing activity. In the current study, the stronger the connectivity strength of the ascending pathway from the bilateral pallidum to the bilateral dLPFC, the stronger the inhibition projecting onto the dLPFC, leading to restrained glutamate release. Buzsáki and Draguhn (2004) demonstrated that the regulation or stabilization of GABA interneurons is critical for the coordination of cortical-mediated behaviors. The dLPFC is activated in emotion tasks and is a main region in managing emotion control (Aupperle et al., 2012). Thus, decreased release of glutamate excitatory neurotransmitter in dLPFC indirectly results in weak emotion management ability.

In addition, gray matter volumes of parts of bilateral caudate, thalamus and putamen in patients with the GG/GT genotype are larger than those with the GG/TT genotype. It was suggested that the T allele in rs3813296 was indirectly associated with inflated volume in these regions. In white matter, the volume of the superior corona radiata fiber in patients with the GG/GT genotype is lower than those with the CG/TT genotype. As mentioned above, the white matter of the corona radiata is the main joint component of the basal ganglia and striatum. Combining the two results regarding gray and white matter, we explained the opposite phenomenon (larger vs. lower) as increscent gray matter volumes (caudate, thalamus, and putamen) extruded contiguous white matter (superior corona radiata fiber) in patients with the GG/GT genotype. This interpretation is inspired by Alliey-Rodriguez et al. (2017).

Until now, we noticed that dLPFC is a key region in the frontal-striatum-thalamus pathway and has the strongest association with other regions. In particular, the causality connections of dLPFC exhibit a significant relationship with clinical behaviors. The current results verified that dLPFC is an important region in the treatment or research of schizophrenia (Olagunju et al., 2017; Schneider et al., 2017).

### Limitations

Two limitations of this study should be considered. First, there was a relatively small sample size in the four subgroups. Currently, gene and neuroimaging data are being collected, and we will replicate and verify these results using a larger sample in the near future. Additionally, the lack of knowledge about the relationships between the region-specific variation in glutamic neurotransmission and temporal patterns of GRIN1 and GRIA2 expression is a common concern to studies of neuroimaging genetics.

## Conclusion

We investigated the main and interaction effects of rs11146020 and rs3813296 on causality connections and structural characteristics in the frontal-striatum-thalamus pathway in Han Chinese patients with schizophrenia. Significant association was found between them, and causality connection strengths affected by two SNPs were remarkably correlated with clinical cognitive performance. Our results suggested that patients with different genotypes have different characteristics, and those patients should receive different clinical interventions.

## Data Availability Statement

The datasets generated in this study is publicly available on the SNP dataset repository: https://www.synapse.org/#!Synapse:syn21788916/tables/, with the following accession no: syn21788916.

## Ethics Statement

The studies involving human participants were reviewed and approved by the Shanghai Mental Health Center Ethics Committee (Serial number: 2012-45). The participants or their legal guardian, provided their written informed consent to participate in this study.

## Author Contributions

SC and YL collected the data. KH and WZ analyzed the data and performed the measurements. SC wrote the manuscript. LH and JW had the major responsibility for preparing the manuscript. QW revised the manuscript.

## Conflict of Interest

The authors declare that the research was conducted in the absence of any commercial or financial relationships that could be construed as a potential conflict of interest.

## Footnotes

1 https://www.synapse.org/

2 http://fsl.fmrib.ox.ac.uk/fsl
